# Tetra methyl bisphenol F: another potential obesogen

**DOI:** 10.1038/s41366-024-01496-5

**Published:** 2024-02-22

**Authors:** Misha Singh, Jennifer Crosthwait, Alexander Sorisky, Ella Atlas

**Affiliations:** 1 Environmental Health Science and Research Bureau (EHSRB), Health Canada, 251 Sir Frederick Banting Driveway, Ottawa, ON K1A 0K9 Canada; 2 Department of Biochemistry, Microbiology and Immunology, University of Ottawa, Ottawa, ON K1H 8M5 Canada; 3 Department of Medicine, University of Ottawa, Ottawa, ON Canada; 4 Chronic Disease Program, Ottawa Hospital Research Institute, Ottawa, ON Canada

**Keywords:** Obesity, Lipids

## Abstract

**Background/Objectives:**

Obesity and its associated metabolic diseases are increasing globally. Sedentary lifestyle, high caloric diet, and genetic predisposition are known to contribute to the onset of obesity. It is increasingly recognized that exposure to environmental chemicals such as Bisphenol A (BPA) may also play a significant role. BPA has been correlated with an array of adverse health effects, including obesity and metabolic disorders. Due to public concern, manufacturers are replacing BPA with structural analogues for which there is limited toxicological data. The objective of this study was to assess the effects of these BPA analogues on adipogenesis.

**Methods:**

The adipogenic effects of Tetra Methyl Bisphenol F (TMBPF), Bisphenol F (BPF), Bisphenol AP (BPAP), and fluorine-9-bisphenol (BHPF) were evaluated in murine 3T3-L1 cells. The cells were treated with BPA and its analogues at concentrations from 0.01 µM to 20 µM, throughout differentiation, in the absence of Dexamethasone (Dex). Lipid accumulation, mRNA and protein levels of adipogenic markers was assessed.

**Results:**

We found that TMBPF, BPF and BPA increased 3T3-L1 lipid accumulation and the expression levels of adipogenic markers lipoprotein lipase (*Lpl*), fatty acid binding protein 4 (*Fabp4*) and perilipin (*Plin*) (1–20 µM; *p* < 0.05), whereas BHPF and BPAP had no effect in this model. Further, TMBPF induced adipogenesis to a greater extent than all the other chemicals including BPA (1–20 µM; *p* < 0.05). The effect mediated by TMBPF on expression levels of *Fabp4*, but not *Plin*, is likely mediated via peroxisome proliferator-activated receptor (PPAR) γ activation.

**Conclusions:**

Of the BPA analogues tested, BPF was most similar to BPA in its effects, while TMBPF was most adipogenic. In addition, TMBPF is likely a PPARγ agonist, it is likely an obesogenic chemical and may be a metabolic disruptor.

## Introduction

The prevalence of obesity has increased worldwide and is now considered an epidemic with 650 million people considered individuals with obesity according to the World Health Organization, 2021 [1]. Obesity is linked to a cluster of metabolic diseases collectively referred to as the metabolic syndrome, including type 2 diabetes and cardiovascular disease [2].

Obesity is a multifactorial condition in which genetics, sedentary life style, and high fat/high caloric diet all contribute to its development [3] and reviewed in [4]. There is also growing evidence that environmental factors may be implicated. The “obesogen hypothesis” was proposed by Grun and Blumberg, defining an obesogen as a xenobiotic that contributes to the development of obesity by increasing adipocyte number, size and/or metabolic function, or hormonal regulation of satiety and appetite [5, 6]. Early exposure to endocrine-disrupting chemicals would predispose individuals to obesity and metabolic complications [5–9]. The introduction of the obesogen hypothesis has since led to the characterization of several chemicals that can be classified as obesogens.

One such chemical is bisphenol A (BPA). BPA was shown to be a potential obesogen in both in vitro and in vivo studies [10, 11]. Human BPA exposure has been correlated with higher rates of metabolic disease, including type 2 diabetes, cardiovascular disease, and liver dysfunction, as well as recurrent miscarriages and an increase in premature births [12–14]. The growing concern over the adverse health effects and the safety of BPA prompted manufacturers to replace BPA with structural analogues in consumer products, such as bisphenol S (BPS), tetra methyl bisphenol F (TMBPF), bisphenol F (BPF), Bisphenol AP (BPAP), and fluorine-9-bisphenol (BHPF) [15–17]. Much of the literature surrounding the toxicity of these analogues remains centred on oestrogenic activity. As such, TMBPF was deemed nontoxic based on the lack of oestrogenic and genotoxic effects [18]. To date, there have not been any in vitro or in vivo studies investigating whether or not BPAP or BHPF may affect adipogenesis, while few recent publications examined the effects of TMBPF and BPF. Our previous studies showed that both BPA and BPS induced adipocyte differentiation and lipid accumulation in murine and human preadipocytes [19, 20]. Further, BPS induced a greater expression of mature adipocyte markers and lipid accumulation compared to BPA in 3T3-L1 preadipocytes [21] and in primary human preadipocytes [19]. A study by Tetzlaff et al. showed that BPF increased lipid accumulation similar to BPA in 3T3-L1 cells, in the presence of both dexamethasone (Dex) and rosiglitazone (ROSI) which are known to induce profound differentiation in this model [22] and another study showed that BPF an BPA increase lipid accumulation and expression of adipogenic markers in Dex free conditions [23]. However, Drobna et al. showed that BPF decreased the expression of adipogenic genes in differentiated 3T3-L1 cells again in the presence of dexamthasone [24]. TMBPF was shown to decrease adipogenesis in human mesenchymal stem cells; however, this study investigated the effects of TMBPF in the presence of treatments that induce maximal adipogenic response in this model [25]. These studies reveal a gap in the literature whereby the effects of BPA analogues on adipogenesis remain unclear and require further investigation.

In the last few decades the 3T3-L1 model was used extensively to identify the molecular mechanism governing the adipogenesis process [26–28] and more recently, it has been used to assess potential obesogens [29]. In the current study, we investigated the effects of four potential BPA replacements in commercial applications - BPAP, BHPF, BPF, and TMBPF - on adipogenesis, in the absence of Dex, but in the presence of iso-butylmethyl xanthine (IBMX) and insulin, using the 3T3-L1 cell model. The combination of insulin, IBMX and Dex induces lipid accumulation in 90–100% of the cells in this model which could obscure any inductive effect of the test chemicals. Adipogenic effects of the chemicals were assessed by quantifying lipid accumulation, as well as mRNA and protein expression levels of adipogenic markers. Our data show that some of the analogues are adipogenic in 3T3-L1 cells and thus they are potential obesogens.

## Materials and methods

### Murine cell culture and preadipocyte differentiation

3T3-L1 mouse embryonic fibroblasts previously obtained from the American Type Culture Collection (ATCC, Manassas, VA, USA) were stored in liquid nitrogen. Cells were thawed and maintained in Dulbecco’s modified Eagle’s medium (DMEM) 5.6 mM glucose media (Hyclone, Mississauga, ON, Canada) supplemented with 1% penicillin/streptomycin (P/S) (Life Technologies, Burlington, ON, Canada) and 10% bovine calf serum (ATCC). Cells were incubated at 37 °C with 10% CO_2_ and left to proliferate. Once 80% confluent, cells were seeded into 6 well plates and left to reach 100% confluence (day −2). Two days post confluence (day 0), cells were differentiated using DMEM medium with 5.5 mM glucose supplemented with 10% foetal bovine serum (FBS; Wisent, Montreal, QC, Canada) along with 1% P/S (Life Technologies, Burlington, ON, Canada).

Differentiation was induced with 0.5 mM IBMX and 100 nM insulin (MI) (Roche Diagnostics, Laval, QC, Canada) with either 250 nM Dex, positive control, or varying concentrations of the following bisphenols; BPA, BPF, TMBPF, BHPF, and BPAP (all from Sigma Aldrich, Oakville, ON, Canada). Solvent controls for these experiments were 0.1% ethanol (for cells treated with Dex) and 0.1% DMSO (for cells treated with 0.01–20 µM BPA, BPF, TMBPF, BHPF, BPAP). Stock concentrations of test chemicals were prepared by reconstituting the lyophilized bisphenols in DMSO at 100 mM stock concentrations. These stocks were then diluted in culture media 1 in 1000 for cell treatment. Dex at 250 µM (1000×) was prepared in ethanol. On day 2 of the differentiation process medium was replaced with 100 nM of insulin alone plus varying concentrations of the bisphenols listed above or solvent control. For Dex only insulin was added from day 2 onwards. Medium was subsequently replenished every two days throughout the differentiation time period (until day 8) and the indicated time points (days 2, 4, 6). For the PPARγ inhibition assay, 2 µM of the PPARγ inhibitor, T0070907 (R&D Systems, MN, USA) was added to the differentiation medium and replenished daily until cells were harvested at day 6 for mRNA extraction. 200 nM Rosiglitazone (ROSI) was used as a positive control for PPARγ inhibition.

### Lipid staining and quantification

3T3-L1 cells were differentiated in a black 96-well plate with clear bottoms (PerkinElmer, Wellesley, MA, USA) for 8 days before being stained. Cells were fixed with 4% paraformaldehyde (Electron Microscopy Sciences, Hatfield, PA, USA) and stained with Nile Red (SigmaAldrich) and 4, 6-diami-dino-2-phenylindole (DAPI) (Sigma Aldrich). Fluorescence readings were taken using the Synergy H4 Hybrid Reader (BioTek, Winooski, VT, USA). Readings were taken at 355/360 (excitation/emission) for DAPI and 485/540 for Nile Red. All data were first normalized to DAPI and then to MI (data represented as fold change over vehicle control). To visualize lipid accumulation within the cells post staining, a Leica SP8 confocal microscope (Leica Microsystems, Toronto, ON, Canada) was used. Photos taken are a representation of the overall lipid accumulation present within the wells.

### RNA extraction and real-time quantitative PCR (RT-qPCR)

3T3-L1 adipocytes were harvested at day 6 during the differentiation process, when the difference in mRNA expression levels of the genes of interest was optimal between the IBMX insulin and vehicle control cells, and the chemical or DEX treated cells. Total RNA was extracted using reagents from the RNeasy Mini Kit (Qiagen, Mississauga, ON, Canada). Genomic DNA was removed using the RNase-Free DNase Kit (Qiagen) as per the manufacturer’s instructions. Expression levels Fatty acid binding protein 4 (*Fabp4*) forward: 5′-GGAAGCTTGTCTCCAGTGAA-3′ reverse: 5′-GCGGTGATTTCATCGAATTC-3′, Lipoprotein Lipase (*Lpl*) forward: 5′-GATCCGAGTGAAAGCCGGAG-3′ reverse: 5′-TTGTTTGTCCAGTGTCAGCCA-3′, Perilipin (*Plin*) forward: 5′-TTGGGGATGGCCAAAGAGAC-3′ reverse: 5′-CTCACAAGGCTTGGTTTGGC-3′, *Adipsin* forward: 5′-CCTGAACCCTACAAGCGATG-3′ reverse: 5′-CAACGAGGCATTCTGGAATA-3′ and *Adiponectin* Forward: 5′-TGACGACACAAAAGGGCTC-3′ Reverse: 5′-CACAAGTTCCCTTGGGTGGA-3′ were quantified using real-time quantitative PCR (qPCR) using *β-actin* Forward: 5′-GACTTCGAGCAAGAGATGGC-3′ Reverse: 5′-CCAGACAGCACTGTGTTGGC-3′ as an internal control. Each reaction consisted of 5 µL SYBR Green Master Mix (Applied Biosystems, Waltham, MA, USA), 0.25 µL of primers (forward and reverse), 0.5 µL of RNase free water, and 4 µL of cDNA. Samples were run in triplicate on 96 well plates (Applied Biosystems) using the CFX96 Real-Time System C1000 Thermal Cycler (BioRad, Hercules, CA, USA) and quantified using the Bio-Rad CFX Software. Data was analyzed using the comparative CT (ΔΔCT) method.

### Human PPARγ reporter assay

PPARγ activation was assessed for BPA, BPF, TMBPF, BHPF and BPAP (0.1,1,10 and 20 µM) using the Human PPARγ Reporter Assay Kit (INDIGO Biosciences, PA, USA) as per the manufacturer’s instructions. Briefly, cells expressing the PPARγ receptor and the luciferase reporter gene were treated for 24 h with bisphenols or the positive control ROSI. Luminescence was quantified in a luminometer plate reader.

### Western blot analysis

Preadipocytes were differentiated as previously described. Cells were lysed at day 8, when protein expression levels of adipogenic genes is optimal in this model, and whole-cell extracts were prepared using lysis buffer containing 50 mM Tris-HCl pH 7.4, 150 mM NaCl, 5 mM EDTA, 0.5% nonidet P-40 and freshly added 1 mM DTT and 1× complete protease inhibitor cocktail (Roche, Basal, Switzerland). Total protein (10–20 μg) was resolved by SDS polyacrylamide gel electrophoresis and transferred to a polyvinylidene difluoride membrane. Membranes were blocked with 5% non-fat milk, and the membranes probed with FABP4, LPL and PLIN antibodies all from Cell Signalling Technologies (Danvers, MA, USA). Membranes were visualized using a ChemiDoc System and quantified using Image Lab software (all from Bio-Rad, Mississauga, ON, Canada).

### Statistical analysis

Delta CT’s were assessed using a blocked reference design in R. Each experimental batch consisted of a control and 5 exposures which defined the blocking effect. The control represents the reference that connects all the experimental conditions. The Anderson-Darling test [30] was used to test the normality assumption using the nortest R package. The Fligner–Killeen test [31] was used to test the homogeneity of variances assumption. The common variance assumption for adiponectin was not satisfied. The rank transformed was applied to adiponectin to investigate the severity of the violation. The untransformed and ranked transformed data resulted in identical conclusions, suggesting that the violation was mild; thus, the untransformed results are presented. Statistical comparisons were conducted using the doBy R package. Results were then exponentiated with base 2 and the delta method was applied to approximate the fold change standard errors. The Sidak method [32, 33] was applied within each family of tests to obtained adjusted *p*-values.

## Results

### BPA TMBPF and BPF induce lipid accumulation in 3T3-L1 preadipocytes

**Fig. 1:**
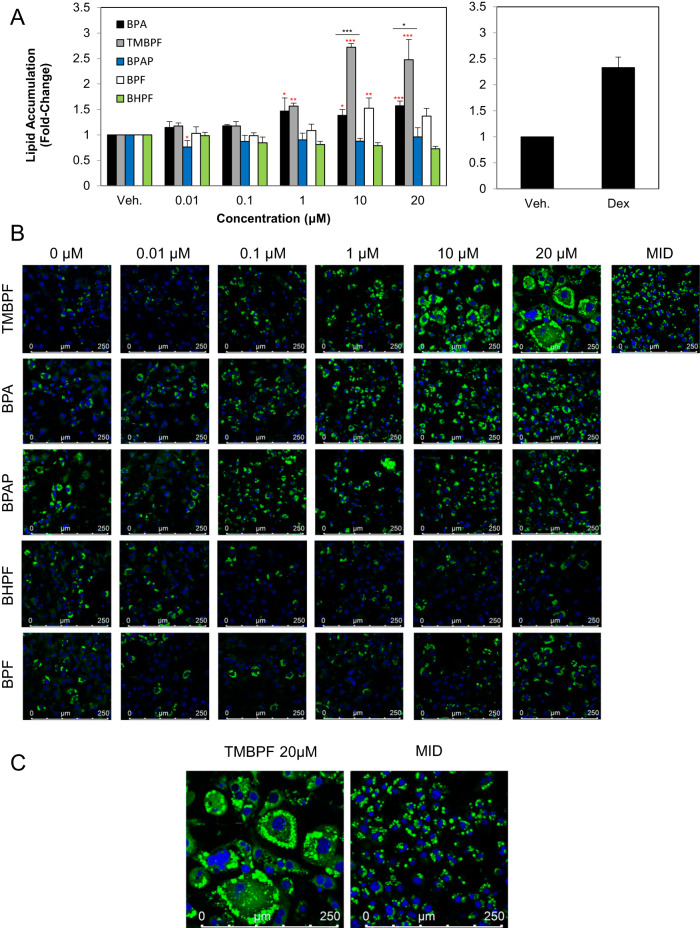
The effects of BPA and its analogues on lipid accumulation in differentiating 3T3-L1 cells. Murine 3T3-L1 preadipocytes were induced to differentiate for 8 days in the presence of 500 µM IBMX, 100 nM insulin, and supplemented with either solvent control (DMSO), TMBPF, BPAP, BHPF, BPF, BPA (0–20 µM), or 250 nM DEX as a positive control (MID). At day 8 of differentiation (**B**) lipid accumulation was visualized using Nile Red (green) staining and then quantified (**A**). **C** Enlarged images of Nile Red stained cells with Dex and 20 µM TMBPF treated cells are also depicted. Lipid accumulation was normalized to DAPI (blue) staining and expressed as fold over DMSO (vehicle) control. Results from at least 3 experiments are graphically represented all chemicals. Data represent mean ± SEM where *P < 0.05, **P < 0.01, ***P < 0.001 was determined relative to vehicle control and *P < 0.05, ***P < 0.001 relative to dose-matched BPA treatment.

To assess the effects of the bisphenols used in this study on lipid accumulation, 3T3-L1 cells were induced to differentiate for 8 days before being stained with DAPI and Nile Red. Lipid accumulation was quantified by fluorescence of Nile Red and normalized DAPI. Analysis of the data revealed increased lipid accumulation with BPA, TMBPF and BPF (Fig. 1A, left panel). BPA induced a 1.5-fold, 1.4-fold and 1.6-fold increase in lipid accumulation at 1, 10 and 20 µM compared to vehicle control. Cells treated with TMBPF showed a 1.56-fold increase at 1 µM, a 2.7-fold increase at 10 µM and a 2.48-fold increase at 20 µM. At 10 µM BPF induced a 1.5-fold increase, while cells treated with Dex induced a 2.3-fold increase (Fig. 1A, right panel). The results show that TMBPF induced lipid accumulation to a greater extent than BPA and to a similar extent as the positive control Dex. BPAP and BHPF had no effect on lipid accumulation in this cell model (Fig. 1A, left panel). No cytotoxicity was observed with any of the chemicals at the concentrations used in this study (data not shown) and as evident by the nuclear staining at the end of differentiation. These results show that TMBPF is as adipogenic as the positive control Dex as measured by lipid accumulation.

### BPA, TMBPF and BPF up-regulate the mRNA levels of adipogenic markers while BHPF displays inhibitory effects. The upregulation of *Fapb4* by TMBPF is mediated via PPARγ activation

**Fig. 2:**
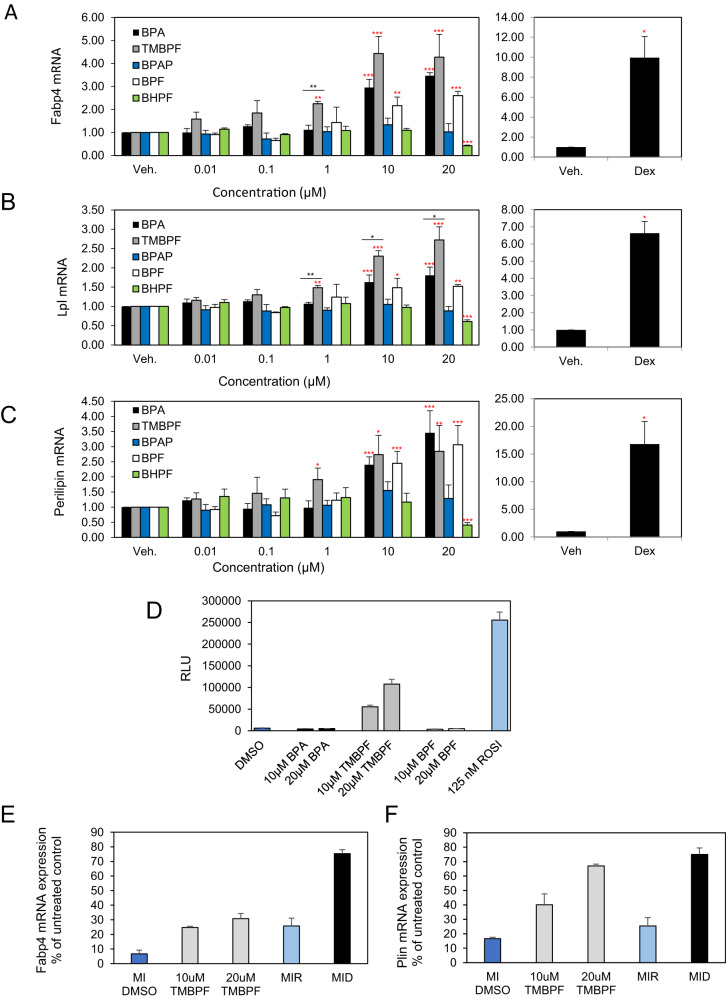
BPA, BPF and TMBPF increase mRNA expression levels of mature adipocytes markers in differentiating 3T3-L1 cells. The upregulation of Fapb4 by TMBPF is mediated via PPARγ activation. **A**-**C** Murine 3T3-L1 preadipocytes were induced to differentiate for 6 days in the presence of 500 µM IBMX, 100 nM insulin, and supplemented with either solvent control (DMSO), BPA, TMBPF, BPAP, BPF, BHPF (0–20 µM) or 250 nM Dex as a positive control. At day 6 of differentiation RNA was isolated and the expression levels of mature adipocyte markers, *Fabp4* (**A**), *Lpl* (**B**) and *Perilipin* (**C**) were quantified by real-time qPCR. Levels were normalized to endogenous *β-actin* levels and expressed as fold over vehicle control. Data represent the mean ± SEM where *P < 0.05, **P < 0.01, ***P < 0.001 relative to vehicle control and *P < 0.05, **P < 0.01 relative to dose-matched BPA treatment. **D** PPARγ activation was assessed with the Indigo human PPARγ reporter assay as per the manufacturers instructions. Cells were treated with the indicated concentrations of bisphenols or Rosiglitazone (ROSI) as a positive control. For PPARγ inhibition, cells were treated daily with 2 µM PPARγ antagonist T0070907. (E) mRNA expression of *Fabp4* and (F) mRNA expression of *Plin* in the presence or absence of inhibitor.

The differentiation process from a preadipocyte to a mature adipocyte is characterized by an increase in mRNA levels of genes that are important for adipocyte function. Therefore, expression levels of mature adipocyte markers *Fabp4*, *Lpl* and *Plin* were assessed at day six of differentiation and are shown in Fig. 2. The results show that BPA increased mRNA expression of *Fabp4* by 3-fold at 10 µM and by 3.46-fold at 20 µM while TMBPF increased its expression by 2.3-fold at 1 µM, 4.4-fold at 10 µM, and by 4.3-fold at 20 µM. BPF also increased *Fabp4* expression levels by 2.2-fold at 10 µM and 2.6-fold at 20 µM (Fig. 2A, left panel). BHPF decreased *Fabp4* expression by 0.4-fold at 20 µM, in the absence of cell cytotoxicity (data not shown). Treatment of the cells with Dex induced a 10-fold increase in *Fabp4* expression levels (Fig. 2A, right panel). BPAP did not affect *Fabp4* expression levels in this model (Fig. 2A, left panel).

*Lpl* mRNA expression was increased by BPA at 10 and 20 μM by 1.6-fold and 1.8-fold, respectively. TMBPF increased mRNA expression of *Lpl* at 1 µM, 10 µM and 20 µM by 1.5-fold, 2.3-fold, and 2.7-fold over vehicle control respectively (Fig. 2B, left panel). BPF increased expression of *Lpl* by 1.5-fold at both 10 and 20 µM. BHPF decreased *Lpl* expression levels by 0.6-fold. Treatment with Dex resulted in a 6.6-fold increase in *Lpl* mRNA expression levels (Fig. 2B, right panel). BPAP had no effect (Fig. 2B, left panel).

**Fig. 3:**
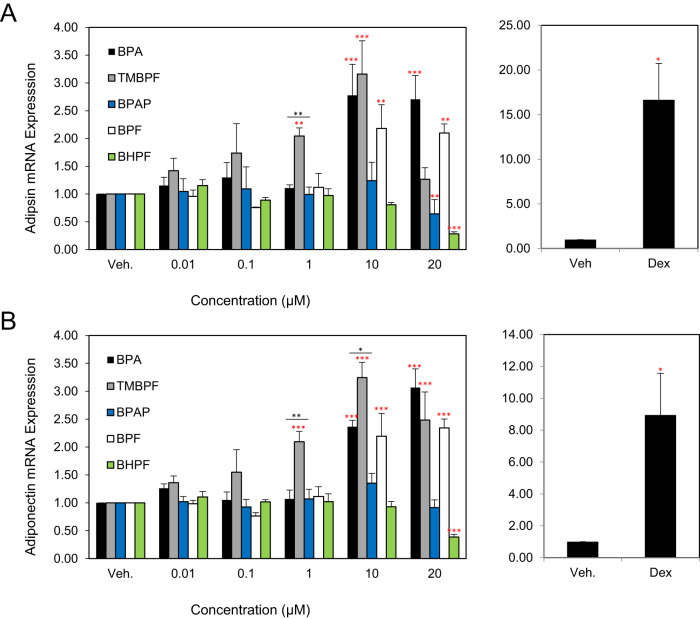
The effects of BPA and its analogues on mRNA expression levels of adipokines in differentiating 3T3-L1 cells. Murine 3T3-L1 preadipocytes were induced to differentiate for 6 days in the presence of 500 µM IBMX, 100 nM insulin, and supplemented with either solvent control (DMSO), BPA, TMBPF, BPAP, BPF, BHPF (0–20 µM) or 250 nM Dex as a positive control. At day 6 of differentiation RNA was isolated and the expression levels of adipokines: *Adipsin* (**A**) and *Adiponectin* (**B**) were quantified by real-time qPCR. Levels were normalized to endogenous *β-actin* levels and expressed as fold over vehicle control. Data represent the mean ± S.E.M where *P < 0.05, **P < 0.01, ***P < 0.001 relative to vehicle control and *P < 0.05, **P < 0.01 relative to dose-matched BPA treatment.

Perilipin mRNA levels were significantly increased by BPA, TMBPF and BPF albeit at different concentrations. BPA induced a 2.4-fold increase at 10 µM and a 3.5-fold increase at 20 µM. While TMBPF elevated mRNA expression of *Plin* by 1.9-fold, 2.7-fold, and 2.8-fold at 1, and 20 µM respectively (Fig. 2C, left panel). BPF induced *Plin* expression level by 2.5-fold at 10 µM and 3-fold at 20 µM. BHPF decreased *Plin* expression levels by 0.4-fold at 20 µM. Dex, increased mRNA expression of *Plin* by 16.8-fold (Fig. 3C, right panel). BPAP had no effect on *Plin* mRNA expression levels (Fig. 3C, left panel). All mRNA expression of the adipogenic markers tested was increased by TMBPF, BPA and BPF, albeit, at different levels. TMBPF was the most potent and increased the expression of these genes to about 50% of the levels induced by Dex. BHPF showed inhibitory effects at the highest concentration.

In order to investigate the mode of action by which the bisphenols exert their effects, we examined whether these chemicals activate nuclear receptors that are known to regulate the adipogenesis process. Our results show that TMBPF, but not BPA or BPF activates PPARγ in a luciferase reporter transactivation assay (Fig. 2D). We also investigated the effect of the BPA analogues on the transcription activity of the glucocorticoid receptor (GR) where there was no observed effect (data not shown). We proceeded also to inhibit PPARγ activity with the PPARγ antagonist T0070907. For this purpose, we treated the cells with the inducers in the presence of the inhibitor and assessed mRNA expression of *Fabp4* and *Plin*. Here we show that *Fabp4* expression was inhibited by 70% for TMBPF and 75% for the PPARγ agonist (ROSI). However, Dex was inhibited only by 25% (Fig. 2E). Interestingly TMBPF effect on *Plin* expression was inhibited only by 30% while ROSI effect was inhibited by 75%. Once again, Dex effect was inhibited by only 25% (Fig. 2F). In summary, the effects of the PPARγ antagonist on TMBPF mediated gene expression seems to be gene specific.

### BPA, TMBPF, and BPF up-regulate the mRNA levels of the adipokines adipsin and adiponectin while BHPF decreases expression levels

The adipose tissue is an endocrine organ that secretes adipokines responsible for the maintenance of the metabolic homoeostasis of the organism. Thus, we investigated the mRNA expression of adipokines *Adipsin* and *Adiponectin*. Increased expression of *Adipsin* was observed with BPA, TMBPF and BPF. BPA increased expression levels by 2.8 and 2.7-fold over vehicle control at 10 and 20 µM, respectively. TMBPF increased expression by twofold and threefold at 1 and 10 µM, respectively. BPF increased expression levels of *Adipsin* by 2-fold at 10 µM and 20 µM. BHPF and BPAP decreased *Adipsin* expression levels at 20 µM by 0.3-fold and 0.6-fold respectively (Fig. 3A, left panel). Dex increased *Adipsin* mRNA expression by 7.5-fold (Fig. 3A, right panel).

The expression level of *Adiponectin* was increased by BPA, TMBPF and BPF. BPA increased *Adiponectin* mRNA expression by 2.4-fold at 10 µM and by 3.0-fold at 20 µM (Fig. 3B, left panel). TMBPF increased *Adiponectin* expression levels by 2-fold at 1 µM, 3.2-fold at 10 µM and 2.5-fold at 20 µM over vehicle control. BPF induced a 2.2-fold increase at 10 µM and a 2.3-fold at 20 µM. BHPF reduced *Adiponectin* expression levels by 0.4-fold at 20 µM. Treatment of cells with Dex increased *Adiponectin* levels by 9-fold over vehicle control (Fig. 3B, right panel). BPAP had no effect (Fig. 3B, left panel). In summary, BPA, TMBPF and BPF increased mRNA expression of both adipokines to similar levels, but to a lesser extent than Dex. BHPF was inhibitory.

### BPA, TMBPF, BPAP and BPF up-regulate the protein expression levels of FABP4 while BHPF decreases its expression

**Fig. 4:**
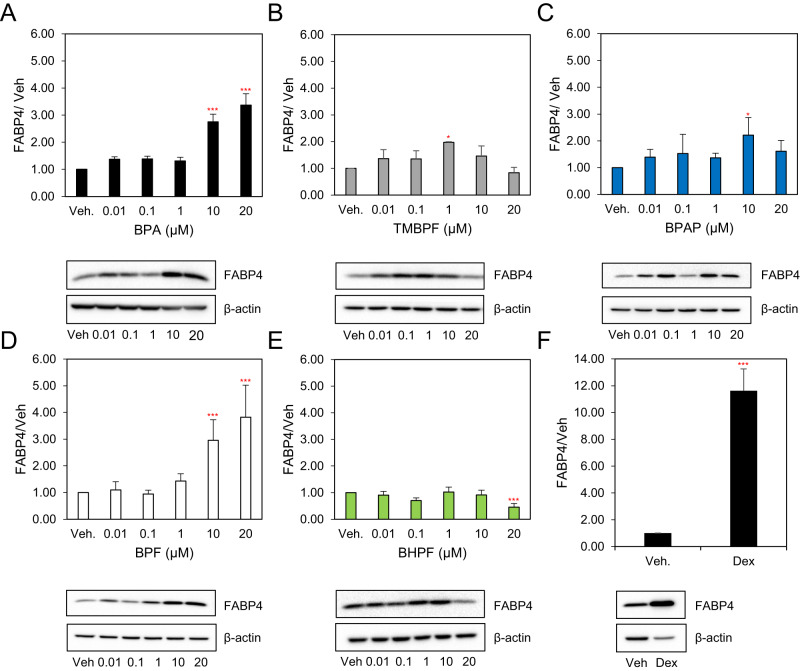
The effects of BPA and its analogues on protein expression levels of FABP4 in differentiating 3T3-L1 cells. Murine 3T3-L1 preadipocytes were induced to differentiate for 8 days in the presence of 500 µM IBMX, 100 nM insulin, and supplemented with either solvent control (DMSO), **A** BPA, **B** TMBPF, **C** BPAP, **D** BPF, **E** BHPF (0–20 µM) or **F** 250 nM Dex as a positive control. At day 8 of differentiation equal amounts of solubilized cellular protein were separated by SDS-PAGE and immunoblotted with the indicated antibodies. β-actin serves as a loading control. Levels were normalized to endogenous β-actin and expressed as fold over DMSO (vehicle) control. Results from 3 experiments are graphically represented. Data represent the mean ± S.E.M where *P < 0.05, **P < 0.01, ***P < 0.001 relative to vehicle control.

To assess the correlation between mRNA expression levels, at day 6, and protein expression at the end of differentiation, cells were induced to differentiate for 8 days and protein expression levels of mature adipocyte markers was evaluated. Protein expression levels of FABP4 were significantly increased by all chemicals except BHPF which decreased expression by 0.45-fold (Fig. 4E). BPA induced a 2.8 and 3-fold increase at 10 and 20 µM respectively (Fig. 4A). Similarly, BPF resulted in a 3-fold increase at 10 µM and a 3.8-fold increase at 20 µM (Fig. 4D). TMBPF increased FABP4 expression by 2-fold at 1 µM (Fig. 4B). Interestingly, BPAP also induced a 2-fold increase in protein expression levels at 10 µM (Fig. 4C). As expected, Dex treated cells exhibited an 11-fold increase compared to vehicle control (Fig. 4F). Protein expression levels correlated with the mRNA expression for BPA, BPF and BHPF, but did not follow the same trend with TMBPF and BPAP treatments.

### BPA, TMBPF, BPAP and BPF up-regulate the protein expression of LPL

**Fig. 5:**
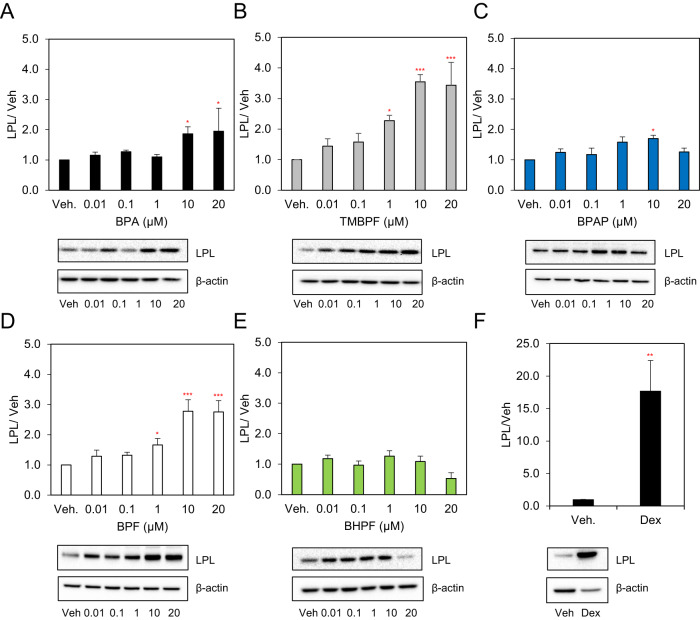
The effects of BPA and its analogues on protein expression levels of LPL in differentiating 3T3-L1 cells. Murine 3T3-L1 preadipocytes were induced to differentiate for 8 days in the presence of 500 µM IBMX, 100 nM insulin, and supplemented with either solvent control (DMSO), **A** BPA, **B** TMBPF, **C** BPAP, **D** BPF, **E** BHPF (0–20 µM) or **F** 250 nM DEX as a positive control. At day 8 of differentiation equal amounts of solubilized cellular protein were separated by SDS-PAGE and immunoblotted with the indicated antibodies. β-actin serves as a loading control. Levels were normalized to endogenous β-actin and expressed as fold over DMSO (vehicle) control. Results from 3 experiments are graphically represented. Data represent the mean ± SEM where *P < 0.05, **P < 0.01, ***P < 0.001 relative to vehicle control.

As expected, BPA increased LPL expression by 2-fold at 10 and 20 µM (Fig. 5A). TMBPF induced expression at 1, 10 and 20 μM by 2.3-fold, 3.5-fold and 3.4-fold, respectively (Fig. 5B). Similarly, BPF increased expression levels of LPL at concentrations as low as 1 µM by 1.7-fold and 2.7-fold at both 10 and 20 μM (Fig. 5D). BPAP treatment resulted in a 1.7-fold increase in LPL at 10 µM despite no effect on lipid accumulation (Fig. 5C). Cells treated with Dex showed a 17.7-fold increase in LPL protein expression levels as compared to control (Fig. 5F). BHPF did not induce LPL expression (Fig. 5E). LPL expression levels correlated with mRNA expression changes for all the bisphenols tested except for BPAP which showed an increase in LPL expression levels at the protein but not at the mRNA level.

### BPA, TMBPF, BPAP and BPF up-regulate the protein expression of PLIN where as BHPF displays inhibitory effects

**Fig. 6:**
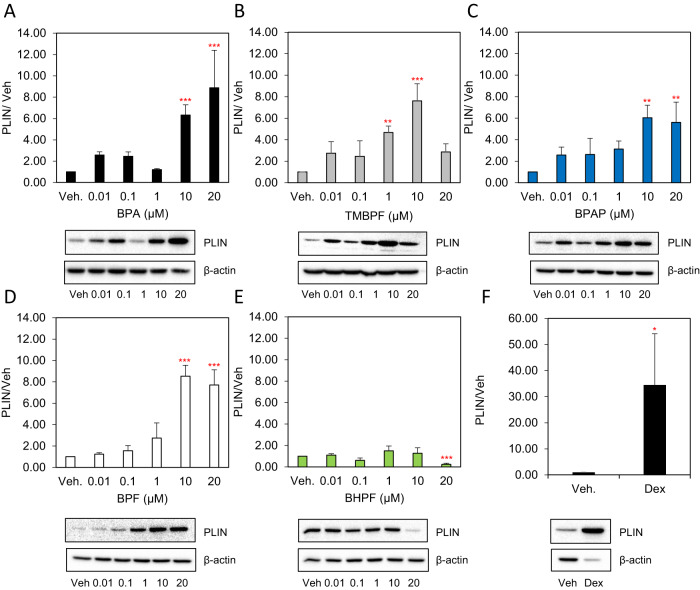
The effects of BPA and its analogues on protein expression levels of PLIN in differentiating 3T3-L1 cells. Murine 3T3-L1 preadipocytes were induced to differentiate for 8 days in the presence of 500 µM IBMX, 100 nM insulin, and supplemented with either solvent control (DMSO), **A** BPA, **B** TMBPF, **C** BPAP, **D** BPF, **E** BHPF (0–20 µM) or **F** 250 nM Dex as a positive control. At day 8 of differentiation equal amounts of solubilized cellular protein were separated by SDS-PAGE and immunoblotted with the indicated antibodies. β-actin serves as a loading control. Levels were normalized to endogenous β-actin and expressed as fold over DMSO (Veh.) control. Results from 3 experiments are graphically represented. Data represent the mean ± S.E.M where *P < 0.05, **P < 0.01, ***P < 0.001 relative to vehicle control.

PLIN protein expression was increased by BPA (Fig. 6A), TMBPF (Fig. 6B), BPAP (Fig. 6C) and BPF (Fig. 6D). BPA induced a 6.3-fold and a 8.9-fold increase at 10 µM and 20 µM, respectively, while TMBPF increased PLIN protein expression by 4.7-fold at 1 µM and 7.6-fold at 10 µM. BPF increased PLIN protein expression by 8.5-fold at 10 µM and by 7.7-fold at 20 µM compared to vehicle control. PLIN protein levels were increased by 6-fold in response to BPAP at 10 µM and 20 µM, despite this chemical’s inability to affect lipid accumulation. Treatment of cells with Dex resulted in a 34.4-fold increase in PLIN protein expression (Fig. 6F). BHPF showed inhibitory effects with a 0.24-fold decrease compared to vehicle control (Fig. 6E).

In summary, PLIN protein expression showed a non-monotonic response upon treatment with BPA. mRNA and protein expression of PLIN in response to BPF and BHPF correlated at all concentrations. Treatment with TMBPF and BPAP showed disconcordant expression of mRNA and protein.

## Discussion

The effects of BPA analogues on human health, and particularly on obesity, remains an area of concern, given that early and continuous exposure to BPA has been linked to many adverse health outcomes, including obesity [34]. Our lab has previously shown that BPA and BPS induced adipogenesis both in murine and human cell models [20, 35]. Here, we investigated the effects of four additional BPA analogues, BPAP, BHPF, BPF and TMBPF. We show that, in addition to BPA, TMBPF and BPF induce adipogenesis in this system. Interestingly, TMBPF was able to increase the lipid in the 3T3-L1 cells to the same extent as the glucocorticoid, Dex, and double the amount of lipid in the BPA-treated cells. Furthermore, the morphology of the cells treated with TMBPF was altered; fewer but larger adipocytes with larger lipid droplets were present in the cultures as compared to the Dex- or other bisphenol-treated cells. Future experiments will investigate these specific phenotypic changes in response to TMBPF.

Previous studies using the 3T3-L1 model showed no significant effect of BPF on adipogenesis and another study showed anti-adipogenic effect with this chemical [22, 24]. Of note, both studies used differentiation media containing Dex, IBMX and insulin. This treatment maximally induces adipogenesis in this model which could obscure any inductive effect of a test chemical acting via a similar or complementary mechanism(s) [36, 37]. Our study investigated the adipogenic potential of BPF in the absence of Dex and we show that BPF was similar to BPA in its effects on adipogenesis under these conditions. A more recent study also showed similar results to the ones presented in this study using the same inducers [23].

TMBPF was more potent than the other bisphenols, including BPA, at increasing lipid accumulation and the expression levels of specific genes at the mRNA level. For example, TMBPF increased *Fabp4* mRNA levels by 4-fold at 10 and 20 µM and by twofold at 1 µM, however, at the protein level, FABP4 was increased only at 1 µM, after which the expression levels dropped back to control levels. Non-monotonic responses have been described for endocrine disrupting chemicals and hormones, such as estradiol, and this may be also true for TMBPF, where effects on FABP4 protein expression may occur at lower concentrations but not at the higher ones [38]. This finding was surprising, and it did not occur with BPA treatment where there was a correlation between the mRNA levels and the protein levels. Since mRNA expression was assessed at day six and protein at day eight it is possible that the timing was not optimal for the protein quantification in the TMBPF treated cells. However, the protein expression levels at day 8 reveal the phenotype of the mature adipocyte that arises as a consequence of chemical treatment. Another possibility is that FABP4 is being secreted by the cells and thus, it is decreased in the cellular lysates. This is especially important because secreted FABP4 was correlated with obesity-related breast cancer [39] and its secretion from adipocytes in individuals with obesity is correlated with adipocyte hypertrophy, harmful effects on other tissues, and insulin resistance [40]. Future research will examine the secreted FABP4 levels in TMBPF-treated cells.

Similarly, TMBPF induced an increase in *Plin* mRNA expression at 1, 10 and 20 µM; however, protein expression levels of PLIN decreased at 20 µM as compared to 1 and 10 µM exposures. At 10 µM, a 7.62-fold increase was observed whereas a 2.86-fold increase was observed at 20 µM. This may seem counterintuitive as lipid accumulation at 10 and 20 μM appear to be relatively the same. We assessed only the expression levels of PLIN1 however, there are other perilipins that can coat the outside of lipid droplets and maintain their integrity [41]. In addition, since the lipid droplets in the 20 µM TMBPF treatment appear to be larger than in the other concentrations and in the Dex-treated cells, the total surface area of the lipid droplets is very likely reduced as compared to many small lipid droplets and thus less PLIN may be required to be present on the surface.

Our results also show that BPAP does not upregulate *Plin* mRNA expression levels, yet there is an increase in its protein expression at 10 and 20 µM. These discrepancies can be attributed to post-transcriptional or translational parameters which may influence the efficiency of protein translation and stability. In addition, mRNA expression, is assessed at a snapshot in time, and thus it is possible that the mRNA levels were not increased yet or that it was degraded already at that time.

In order to gain further insight as to the effects of the bisphenol analogues, we examined the expression of the adipokines *Adipsin*, and *Adiponectin*. As expected, we observed that the expression of the adipokines was increased in cells treated with BPA, BPF, and TMBPF. However, we also noted a significant decrease in the expression of *Adipsin* from 3.1-fold at 10 µM to control levels at 20 µM in cells treated with TMBPF, despite high levels of differentiation in those cells. Not much is known about the regulation of adipsin; however, in vivo mouse studies have shown a correlation between reduced mRNA expression of *Adipsin* in adipocytes of obese mice as compared to lean mice [42]. This study also reported that decreased levels of adipsin in the adipose tissue of obese mice was correlated with elevated endoplasmic reticulum (ER) stress markers as compared to lean mice, and ER stress was correlated with insulin resistance and inflammation of adipose tissue of individuals with obesity [42, 43]. It is possible that TMBPF induces ER stress at the highest concentration as shown for other chemicals such as BPA and MEHP [44]. Another study showed that BPA, at low doses, increased metabolic dysfunction and inflammatory cytokine expression in adipose tissue of mice on high fat diet [45] and thus it is possible that TMBPF has similar effects.

Cells treated with TMBPF had equal amounts of lipid as the cells treated with the optimal differentiation cocktail, IBMX, Insulin, and Dex. However, the expression of adipokines was different. Dex-treated cells had a 9-fold and 16-fold increased expression of *adiponectin* and *adipsin* as compared to vehicle control, respectively, while cells treated with TMBPF only increased the expression by threefold for both adipokines. The cells treated with TMBPF were also larger with bigger lipid droplets as compared to Dex treated cells. Of note, this phenotype is similar to that of hypertrophied adipocytes, which occurs in obesity. It is also worth mentioning that hypertrophied adipocytes secrete less adiponectin [46], which seems to be also true for the cells differentiated in response to TMBPF. This may indicate that TMBPF exposure may lead to dysfunctional adipocytes which in turn may suggest that TMBPF is a metabolic disruptor.

In order to gain some insight as to the mechanism of action of the adipogenic chemicals, we performed transcription assays targeting the PPARγ nuclear receptor. TMBPF appears to be a potent activator of PPARγ as shown by a luciferase assay and by the ability of a PPARγ antagonist to inhibit the TMBPF mediated response. Interestingly, BPF and BPA appear to mediate their adipogenic effect via a different mechanism since they could not activate PPARγ. Also of interest, is the gene specific response to TMBPF where *Fabp4* expression was inhibited with the PPARγ antagonist similar to ROSI, the PPARγ agonist. However, *Plin* expression was not affected greatly by the antagonist in the presence of TMBPF. On the other hand, *Plin* expression as a result of treatment with ROSI was inhibited. Of note, both genes were not downregulated greatly by the PPARγ antagonist in the MID treatment. This shows that there are compensatory mechanisms that may be at play when PPARγ is being inhibited.

This is the first study to show that the BPAP and BHPF do not induce adipogenesis in 3T3-L1 preadipocytes. In fact, BHPF down-regulated the expression levels of all adipogenic markers tested in the cells treated with IBMX and insulin. It is possible that BHPF inhibits either the insulin pathway or the cyclic AMP pathway, as general cytotoxicity was not a factor with any of the bisphenol tested. In fact, adiponectin was downregulated by BHPF and it is downstream of the insulin pathway [47]. Unlike the other BPA analogues, BPAP affected the expression levels of specific adipogenic makers at the protein level without affecting adipogenesis. For example, BPAP increased the protein expression levels of PLIN by 6-fold compared to control. It is possible that PLIN is present in the cell but not bound to lipid droplets since we did not observe an increase in lipid droplet formation in response to this chemical [41].

One of the limitation of this study is the use of micromolar concentrations. Exposure to some of the BPA analogues has been determined and ranges between 0.15–1.5 ng/mL for BPA, 0.09–0.2 ng/mL for BPS, and 0.01–0.12 ng/mL for BPF [48]. To the best of our knowledge, exposure to TMBPF has not yet been evaluated, however it is expected to increase as it has been deemed as one of the safe replacements for BPA [49]. In vitro studies commonly rely on high concentrations for the observed effects [21, 50, 51]. Future studies need to address the amount of the intracellular concentrations of the compounds used as they can adhere to the plastic and or to the proteins in the culture media. In vivo studies often observe effects at much lower doses of the chemicals such as in the case of BPA where metabolic disruption was observed in mice at low concentrations [52]. Thus justifying the relevance of our study as these chemicals may induce effects at much lower concentrations in vivo.

In summary, of the BPA analogues that we evaluated, TMBPF was the most adipogenic of all bisphenols including BPA. Data on the toxicological effects of TMBPF are few and are mainly limited to the oestrogenic effects of the chemical. TMBPF has been deemed a safe BPA replacement based on the lack of oestrogenic activity [18]. This is the first study to investigate the effects of TMBPF on adipogenesis in 3T3-L1 cells. Our results show that TMBPF increased lipid accumulation to a greater extent than all the other bisphenols tested and exhibited similar potency to Dex, the positive control. Also our results show that TMBPF is a potent activator of PPARγ and exerts at least some of its adipogenic effects through this receptor. Thus, based on this study TMBPF is a potential obesogen and may be a metabolic disruptor.

## Data Availability

All data are available upon request.
